# Identification and management of *Shigella* infection in children with diarrhoea: a systematic review and meta-analysis

**DOI:** 10.1016/S2214-109X(17)30392-3

**Published:** 2017-11-10

**Authors:** Kirkby D Tickell, Rebecca L Brander, Hannah E Atlas, Jeffrey M Pernica, Judd L Walson, Patricia B Pavlinac

**Affiliations:** a Department of Global Health, University of Washington, Seattle, WA, USA; b Department of Epidemiology, University of Washington, Seattle, WA, USA; c Department of Pediatrics, University of Washington, Seattle, WA, USA; d Department of Medicine, University of Washington, Seattle, WA, USA; e Division of Infectious Disease, Department of Pediatrics, McMaster University, Hamilton, ON, Canada

## Abstract

**Background:**

*Shigella* infections are a leading cause of diarrhoeal death among children in low-income and middle-income countries. WHO guidelines reserve antibiotics for treating children with dysentery. Reliance on dysentery for identification and management of *Shigella* infection might miss an opportunity to reduce *Shigella*-associated morbidity and mortality. We aimed to systematically review and evaluate *Shigella*-associated and dysentery-associated mortality, the diagnostic value of dysentery for the identification of *Shigella* infection, and the efficacy of antibiotics for children with *Shigella* or dysentery, or both.

**Methods:**

We did three systematic reviews (for mortality, diagnostic value, and antibiotic treatment of *Shigella* and dysentery), and meta-analyses where appropriate, of studies in resource-limited settings. We searched MEDLINE, Embase, and LILACS database for studies published before Jan 1, 2017, in English, French, and Spanish. We included studies of human beings with diarrhoea and accepted all study-specific definitions of dysentery. For the mortality and diagnostic value searches, we excluded studies that did not include an effect estimate or data necessary to calculate this estimate. The search for treatment included only randomised controlled trials that were done after Jan 1, 1980, and assessed antibiotics in children (aged <18 years) with dysentery or laboratory-confirmed *Shigella*. We extracted or calculated odds ratios (ORs) and 95% CIs for relative mortality and did random-effects meta-analysis to arrive at pooled ORs. We calculated 95% CIs assuming a binomial distribution and did random-effects meta-regression of log-transformed sensitivity and specificity estimates for diagnostic value. We assessed the heterogeneity of papers included in these meta-analyses using the *I*^2^ statistic and evaluated publication bias using funnel plots. This review is registered with PROSPERO (CRD42017063896).

**Findings:**

3649 papers were identified and 60 studies were included for analyses: 13 for mortality, 27 for diagnostic value, and 20 for treatment. *Shigella* infection was associated with mortality (pooled OR 2·8, 95% CI 1·6–4·8; p=0·000) whereas dysentery was not associated with mortality (1·3, 0·7–2·3; p=0·37). Between 1977 and 2016, dysentery identified 1·9–85·9% of confirmed *Shigella* infections, with sensitivity decreasing over time (p=0·04). Ten (50%) of 20 included antibiotic trials were among children with dysentery, none were placebo-controlled, and two (10%) evaluated antibiotics no longer recommended for acute infectious diarrhoea. Ciprofloxacin showed superior microbiological, but not clinical, effectiveness compared with pivmecillinam, and no superior microbiological and clinical effectiveness compared with gatifloxacin. Substantial heterogeneity was reported for meta-analyses of the *Shigella*-associated mortality studies (*I*^2^=78·3%) and dysentery-associated mortality studies (*I*^2^=73·2%). Too few mortality studies were identified to meaningfully test for publication bias. No evidence of publication bias was found in this analysis of studies of diagnostic value.

**Interpretation:**

Current WHO guidelines appear to manage dysentery effectively, but might miss opportunities to reduce mortality among children infected with *Shigella* who present without bloody stool. Further studies should quantify potential decreases in mortality and morbidity associated with antibiotic therapy for children with non-dysenteric *Shigella* infection.

**Funding:**

Bill & Melinda Gates Foundation and the Center for AIDS Research International Core.

## Introduction

In resource-limited settings, *Shigella* species (*Shigella*) are a leading cause of childhood diarrhoea^1^, ^2^ and have case-fatality rates of up to 28% in children with severe disease.^3^, ^4^ The manifestations of *Shigella* can include watery diarrhoea, dysentery, and complications such as encephalopathy.^5^, ^6^ WHO diarrhoea guidelines^7^, ^8^ focus on rehydration, and the provision of zinc, and they specifically address *Shigella* infections by recommending ciprofloxacin be given to children with dysentery, defined as observed presence or caregiver report of blood in the patient's stool. Stool culture is unavailable in many resource-limited settings; therefore, this recommendation is based on evidence showing a strong association between *Shigella dysenteriae* type 1 and dysentery, and the documented efficacy of antibiotics for treating dysenteric *Shigella*.^9^, ^10^, ^11^ However, substantial mortality and morbidity are observed in children with non-dysenteric *Shigella* infection and these children might benefit from prompt antibiotic treatment.

**Research in context**

**Evidence before this study**

We did a preliminary literature search of MEDLINE in October, 2015, using the search terms “dysentery” and “*Shigella*”. This search revealed studies from sub-Saharan Africa where a large proportion of children infected with *Shigella* do not present with dysentery. However, WHO guidelines recommend antibiotics only for children presenting with dysentery, unless cholera is suspected. Restriction of antibiotic treatment to these children is a pragmatic stewardship measure in settings where diagnostics are rarely available, but might result in an appreciable amount of unaddressed *Shigella*-associated morbidity and mortality.

**Added value of this study**

Through systematic reviews and meta-analyses of evidence available form MEDLINE, Embase, and LILACs database, this study suggests that current international guidelines might not be addressing the full burden of *Shigella*-associated mortality.

**Implications of all the available evidence**

Clinicians should continue to aggressively manage dysentery, but should be aware that the absence of dysentery does not indicate a low risk of death and does not exclude *Shigella* as a cause of diarrhoea. In particularly vulnerable children with diarrhoea, such as those younger than 2 years or with malnutrition, identification and treatment of *Shigella* infection might be life-saving. Clinical trials are urgently needed to support the development of management guidelines for non-dysenteric *Shigella* infections.

The *Shigella* genus includes four species—*S dysenteriae, S sonnei, S flexneri*, and *S boydii—*and unique serotypes, such as *S dysenteriae* type 1. These species vary in their tendency to cause dysentery. *S dysenteriae* type 1 and, to a lesser extent, *S flexneri* are most strongly associated with bloody stool.^12^ However, recent studies have shown a global decline in the incidence of *S dysenteriae* type 1, which can cause epidemic or pandemic dysentery. In the Global Enteric Multicenter Study^12^ of 9439 children with moderate-to-severe diarrhoea in seven countries between 2007 and 2011, no cases of *S dysenteriae* type 1 were identified, and this serotype was not identified in 56 958 diarrhoeal episodes recorded in another multicountry study.^13^ Surveillance data from Bangladesh have not documented a case of *S dysenteriae* type 1 infection since 2005, while the prevalence of other *Shigella* serotypes has remained relatively constant.^14^ This change in species prevalence might result in fewer children with *Shigella* infection presenting with bloody stool; therefore, fewer children might receive antibiotic treatment with current guidelines.

Because of the ongoing contribution of *Shigella* to childhood diarrhoeal morbidity and mortality, and the changing epidemiology of *Shigella* globally, we aimed to examine evidence supporting dysentery-based *Shigella* management and to systematically review the available literature to assess associations between symptomatic *Shigella* infection, dysentery, and death. We also aimed to examine the diagnostic value of dysentery for identifying individuals infected with *Shigella* and the efficacy of antibiotics for children with *Shigella* or dysentery, or both.

## Methods

### Search strategy and selection criteria

We did systematic searches using MEDLINE, Embase, and LILACS database for studies published before Jan 1, 2017. The first search focused on *Shigella*-associated and dysentery-associated mortality (mortality), the second search focused on the use of dysentery as a marker of *Shigella* infection (diagnostic value), and the third search focused on the treatment of *Shigella* infections and dysentery (treatment). The appendix (p 1) shows the full list of search terms used. We considered in all three searches papers published in English, French, and Spanish that reported data from low-income or middle-income countries (as defined by the World Bank, June, 2015), and we only included studies of human beings with diarrhoea. We accepted all study-specific definitions of dysentery, including maternal report of blood with or without mucus in the stool or direct observation at diarrhoea presentation.

For the mortality search, we included studies of any design that reported associations between *Shigella* or dysentery and mortality, or the case fatalities of different *Shigella* species; and we excluded studies that did not include an effect estimate or data necessary to calculate this estimate. In the diagnostic value search, we included studies of any design from which the proportion of participants with laboratory-confirmed *Shigella* infections and dysentery (sensitivity) or the proportion of children not infected by *Shigella* and without dysentery (specificity) could be extracted. We included studies of adults and children in both mortality and diagnostic value searches. Lastly, we limited the treatment search to trials after Jan 1, 1980, and to children younger than 18 years, and we included studies with titles and abstracts that contained the terms *Shigella*, shigellosis, dysentery, or blood in stool. We only included randomised controlled trials of one or more antibiotics among children with dysentery or laboratory-confirmed *Shigella* infections.

Titles and abstracts of eligible studies were independently reviewed by two authors (KDT, PBP, or RLB). If authors disagreed on inclusion, consensus was reached following full-text review. Data were extracted from included studies by a single author (KDT or RLB).

### Data analysis

We identified all duplicate data by comparing the study population, sample sizes, and enrolment dates of eligible studies and removed them before analysis. For eligible studies of mortality, we extracted odds ratios (ORs) and 95% CIs for relative mortality. We calculated ORs and 95% CIs using data extracted from the publications or provided by the paper's corresponding author and did random-effects meta-analysis to arrive at pooled ORs. We assessed the quality of individual studies using modified Grading of Recommendations Assessment, Development and Evaluation (GRADE) criteria^15^ (appendix p 2). In eligible studies of diagnostic value, we calculated 95% CIs assuming a binomial distribution. We did random-effects meta-regression of log-transformed sensitivity and specificity estimates (proportions) by the middle year of study enrolment to identify a possible time-trend in sensitivity and specificity estimates. We assessed the quality of included studies using the Quality Assessment of Diagnostic Accuracy Studies criteria^16^ (appendix p 3). Lastly, for eligible studies of treatment, we summarised clinical, anthropometric, and microbiological outcomes, and assessed the quality of included trials using modified GRADE criteria^15^ (appendix p 4). The appendix (p 1) summarises all the variables extracted for each search.

We used Stata (version 13.1) for all analyses. We assessed the heterogeneity of papers included in these meta-analyses using the *I*^2^ statistic and evaluated publication bias using funnel plots (appendix p 6). This review is registered with PROSPERO (CRD42017063896).

### Role of the funding source

The funder of the study had no role in study design, data collection, data analysis, data interpretation, or writing of the report. The corresponding author had full access to all the data in the study and had final responsibility for the decision to submit for publication.

## Results

**Figure 1:**
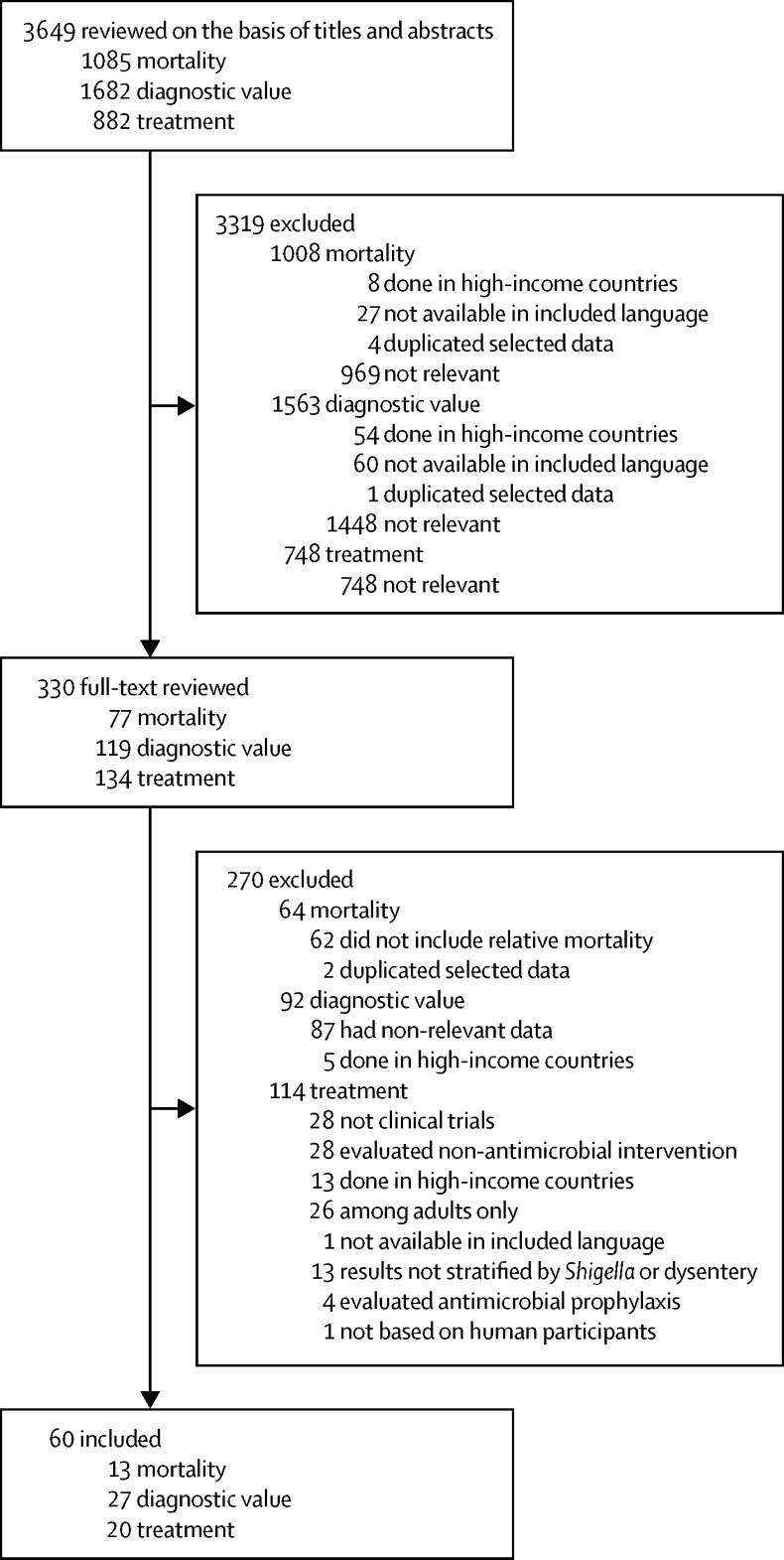
Study selection profile

For the mortality search, 1085 titles and abstracts were screened and 13 studies met inclusion criteria (figure 1). The enrolment period of included studies ranged from 1974 to 2013, and 11 (85%) of 13 studies ascertained inpatient deaths only. Eight (62%) were done in Asia, with seven in Bangladesh. Three (23%) were done in sub-Saharan Africa, one (8%) in Turkey, and one (8%) was a multi-site study. Nine (69%) studies included the relative mortality of *Shigella* (n=seven) or dysentery (n=six), all of which used children with other causes or presentations of diarrhoea as a reference group (table 1).

**Table 1:** Odds of death associated with culture-confirmed *Shigella* spp or dysentery at diarrhoea presentation as compared with children without *Shigella* infection or dysentery

	**Enrolment dates**	**Population and study characteristics**	**N**	**Number of dysentery cases (deaths)**	**Number of *Shigella* cases (deaths)**	**Dysentery OR**	***Shigella* OR**
Dutta et al (1995)^17^	1990	Inpatients aged <5 years with acute watery diarrhoea, persistent diarrhoea, or dysentery; study done in India; dysentery defined as ≥3 loose stools with blood and mucus on caregiver report; *Shigella* spp detected by culture; *Shigella* spp and dysentery compared with other diarrhoeal admission; deaths ascertained during admission to hospital	380	75 (16)	53 (22)	1·7 (0·8–3·3)	5·4 (2·7–10·6)*
Kotloff et al (2013)^1^	2007–11	Children aged 12–23 months with moderate-to-severe diarrhoea; study done in multiple countries; dysentery defined as visible bloody stool; *Shigella* spp detected by culture and compared with children with diarrhoea who were negative for *Shigella* spp; deaths ascertained within 90-day follow-up (in and out of hospital)	3205	..	485 (8)	..	0·9 (0·4–1·8)
Islam et al (1986)^4^	1980–81	Inpatients of all ages with diarrhoea; study done in Bangladesh; *Shigella* spp detected by culture and compared with other diarrhoeal admission; deaths ascertained during admission to hospital	3251	..	436 (75)	..	1·6 (1·2–2·1)*
O'Reilly et al (2012)^3^	2005–07	Inpatients <5 years with watery, mucoid, or bloody diarrhoea; study done in Kenya; dysentery defined as visible bloody stool; *Shigella* spp detected by culture; *Shigella* spp and dysentery compared with other diarrhoeal admission; deaths ascertained during admission to hospital	1146	96 (10)	42 (12)	1·1 (0·6–2·3)	4·2 (2·1–8·5)*
Pernica et al (2016)^18^	2011–13	Children aged <13 years admitted to hospital with diarrhoea in Botswana; dysentery defined as bloody diarrhoea on caregiver report; *Shigella* spp detected by PCR; *Shigella* spp and dysentery compared with other diarrhoeal admission; deaths ascertained during admission to hospital	671	74 (4)	109 (7)	1·4 (0·5–4·3)	1·9 (0·8–4·6)
Ronsmans et al (1988)^9^	1986	Community members of all ages with watery, mucoid, or bloody and mucoid diarrhoea; study done in Bangladesh; dysentery defined as visible bloody mucoid stool and compared with children with watery or mucoid-bloodless diarrhoea; length of follow-up period not specified, but captured deaths in hospitals and in the community	46 607	17 953 (122)	..	2·6 (1·9–3·5)*	..
Teka et al (1996)^19^	1990–94	Inpatients aged <5 years with diarrhoea; study done in Bangladesh; *Shigella* spp detected by culture and compared with other diarrhoeal admission; deaths ascertained during admission to hospital	184	..	24 (14)	..	5·6 (2·3–13·8)*
Uysal et al (2000)^20^	1995–97	Inpatients aged 1 month to 5 years with diarrhoea, mucoid diarrhoea, or bloody diarrhoea; study done in Turkey; unclear definition of dysentery; *Shigella* spp detected by culture; *Shigella* spp and dysentery compared with other diarrheal admission; deaths ascertained during admission to hospital	400	NA†	21 (5)	0·6 (0·1–3·1)	5·1 (1·3–16·2)*
van den Broek et al (2005)^21^	1993–99	Severely malnourished inpatients‡ aged <4 years with diarrhoea; study done in Bangladesh; unclear definition of dysentery; *Shigella* spp detected by culture; dysenteric *Shigella* compared with dysentery-negative *Shigella*; deaths ascertained during admission to hospital	200	66 (28)	200 (100)	0·6 (0·4–1·2)	..

The appendix (p 2) summarises the associated GRADE quality assessment. GRADE=Grading of Recommendations Assessment, Development and Evaluation. OR=odds ratio. NA=not available. WAZ=weight-for-age Z score.

* p<0·05.

† Number of children with dysentery not reported.

‡ Severe malnutrition was defined using Gomez classification WAZ <60% of National Center for Health Statistics median.

1682 titles and abstracts were reviewed and 27 studies were included from the diagnostic value search (figure 1). 13 (48%) of 27 studies were done in Asia, seven (26%) in Africa, five (19%) in the Middle East, and two (7%) in Latin America. Dysentery was assessed by visual inspection at presentation (14 [52%] of 27 studies), caregiver report (three [11%]), visual confirmation or reported history (six [22%]), or not described (four [15%]). The sensitivity of dysentery for identification of *Shigella* infection was extracted from all 27 studies whereas specificity was available in 20 (74%) studies (table 2).

**Table 2:** Sensitivity and specificity of dysentery at diarrhoea presentation for the identification of *Shigella* infection in children

	**Enrolment dates**	**Population and study characteristics**	**Number of children with *Shigella* (*Shigella* with dysentery)**	Number of children with dysentery*	**Sensitivity (95% CI)**	**Specificity (95% CI)**
Pavlinac et al (2016)^22^	2010–14	1360 outpatients aged 6 months to 5 years with acute diarrhoea; study done in Kenya; observation of bloody stool by laboratory technician and history of bloody stool by caregiver report used to indicate dysentery	63 (7)	86	11·1% (4·6–21·6)	94·0% (92·6–95·2)
Pernica et al (2016)^18^	2011–13	671 children aged <13 years presenting to hospital with diarrhoea; study done in Botswana; history of bloody stool by caregiver report used to indicate dysentery	109 (29)	74	26·6% (23·2–30·0)	92·0% (89·4–94·1)
Aggarwal et al (2016)^23^	2011–12	385 children aged <12 years presenting to hospital with diarrhoea; study done in India; history of bloody stool by caregiver report or clinician observation used to indicate dysentery	56 (39)	118	69·6% (55·9–81·2)	64·4% (58·4–69·7)
Eseigbe et al (2013)^24^	2011	270 children aged <5 years presenting to hospital with diarrhoea who had a stool culture; study done in Nigeria; unclear definition of dysentery	9 (5)	28	55·6% (21·2–86·3)	91·1% (87·1–94·3)
Hegde et al (2013)^25^	2007–12	3399 children aged <5 years presenting to facilities with diarrhoea; study done in Guatemala; unclear definition of dysentery	261 (5)	..	1·9% (0·6–4·4)	..
Dooki et al (2014)^26^	2009	172 children aged <14 years referred to hospital for acute diarrhoea or dysentery; study done in Iran; unclear definition of dysentery	7 (4)	33	57·1% (18·4–90·1)	82·4% (75·7–87·9)
Debas et al (2011)^27^	2009	215 inpatients of all ages with watery, bloody, or mucoid diarrhoea; study done in Ethiopia; observation of bloody stool by laboratory technician used to indicate dysentery	32 (9)	39	28·1% (13·7–46·7)	83·6% (77·4–88·7)
El-Shabrawi et al (2015)^28^	2007–09	356 children aged <5 years admitted with acute diarrhoea; study done in Egypt; dysentery defined as visible blood in stool; history of bloody stool by caregiver report used to indicate dysentery	4 (2)	69	50·0% (6·8–93·2)	81·0% (76·5–87·9)
Jafari et al (2008)^29^	2004–05	808 inpatients of all ages with acute diarrhoea; study done in Iran; observation of bloody stool by unspecified observer used to indicate dysentery	155 (39)	111	25·2% (18·5–32·8)	89·0% (86·3–91·3)
Ozmert et al (2010)^30^	2003–06	130 inpatients aged 1–16 years with gastroenteritis whose stool contains blood, mucus, or neither; study done in Turkey; observation of bloody stool (unspecified observer) used to indicate dysentery	65 (19)	19	29·2% (18·6–41·8)	100% (94·8–100)
von Seidlein et al (2006)^13^	2000–04	51 826 Individuals of all ages presenting to community clinics or district hospitals with diarrhoea or dysentery (≥one loose bowel movement with visible blood); study done in Bangladesh, China, Pakistan, Indonesia, Vietnam, and Thailand; observation of bloody stool by unspecified observer used to indicate dysentery	2925 (790)	4751	27·0% (24·4–28·6)	92·7% (92·4–92·9)
van den Broek et al (2005)^21^	1993–99	200 severely malnourished† inpatients aged <4 years with diarrhoea and culture-confirmed *Shigella dysenteriae* type 1 or *Shigella flexneri;* study done in Bangladesh; history of visible blood in stool was used to indicate dysentery	200 (66)	..	33·0% (26·5–40·0)	..
Suwatano et al (1997)^31^	1995–96	106 inpatients aged 1 month to 5 years with acute diarrhoea; study done in Thailand; observation of bloody and mucoid stool by unspecified observer used to indicate dysentery	8 (3)	12	37·5% (31·7–44·6)	90·8% (83·3–95·7)
Youssef et al (2000)^32^	1994–95	265 inpatients aged <5 years with acute diarrhoea; study done in Jordan; observation of bloody stool by clinician used to indicate dysentery	10 (6)	28	60·0% (36·1–80·9)	91·4% (87·3–94·5)
Nakano et al (1998)^33^	1992–93	639 inpatients aged <5 years; study done in Zambia; observation of bloody diarrhoea by unspecified observer used to indicate dysentery	65 (51)	220	78·5% (66·5–87·7)	70·6% (66·6–74·3)
Mathan et al (1991)^34^	1989–90	916 inpatient and community-based infants and children aged <3 years with acute diarrhoea or dysentery, or both; study done in India; observation of bloody stool by unspecified observer used to indicate dysentery	152 (94)	191	61·8% (53·6–69·6)	87·3% (84·7–89·6)
Sobel et al (2004)^35^	1989–90	414 inpatients aged 1–5 years with acute diarrhoea; study done in Brazil; observation of bloody stool by unspecified observer used to indicate dysentery	66 (35)	39	53·0% (48·0–57·8)	98·9% (91·7–100)
Khan et al (2013)^6^	1987–89	792 inpatients aged <15 years with diarrhoea and culture-confirmed *Shigella* spp; study done in Bangladesh; history of bloody stool as indicated in patient record and observation by caregiver used to indicate dysentery	792 (332)	..	41·9% (38·6–45·6)	..
Ahmed et al (1997)^36^	1987–89	1756 community-based children aged <5 years with diarrhoea or dysentery (diarrhoea described as bloody); study done in Bangladesh; history of bloody diarrhoeal episodes by caregiver report at enrolment or any time during 31 days of follow-up used to indicate dysentery	219 (86)	313	39·3% (32·8–46·1)	85·2% (83·4–87·0)
Kagalwalla et al (1992)^37^	1985–90	229 inpatients aged <13 years with diarrhoea, haematochezia, or abdominal pain and culture-confirmed *Shigella* spp; study done in Saudi Arabia; observation of bloody stool by unspecified observer used to indicate dysentery	229 (86)	..	37·6% (31·7–44·6)	..
Dutta et al (1992)^38^	1985–88	950 inpatients aged <5 years with culture-confirmed *Shigella*; study done in India; observation of bloody and mucoid stool by unspecified observer used to indicate dysentery	192 (165)	..	85·9% (80·2–90·5)	..
Echeverria et al (1991)^39^	1986–87	471 inpatients <5 years with diarrhoea and culture-confirmed *Shigella* spp, *Salmonella* spp, *Campylobacter* spp, diarrhoeagenic *Escherichia coli*, or rotavirus; study done in Thailand; observation of bloody stool by unspecified observer used to indicate dysentery	94 (37)	110	39·4% (29·4–50·0)	80·6% (76·3–84·5)
Moalla et al (1994)^40^	1986	170 children aged <6 years presenting with acute diarrhoea; study done in Tunisia; unclear definition of dysentery	14 (8)	..	57·1% (28·9–82·3)	..
Huskins et al (1994)^41^	1984–88	318 inpatients (159 aged <3 months and 159 aged 1–10 years) with culture-confirmed *Shigella* spp; study done in Bangladesh; observation of bloody stool by unspecified observer used to indicate dysentery	318 (117)	..	36·8% (28·0–46·2)	..
Ronsmans et al (1988)^9^	1984	300 community members of all ages, with watery, mucoid, or bloody diarrhoea; study done in Bangladesh; observation of bloody stool by medical assistant or history of bloody stool by caregiver report used to indicate dysentery	82 (51)	80	62·2% (50·8–72·7)	86·7% (81·5–91·0)
Stoll et al (1982)^42^	1979–80	3550 inpatients of all ages with acute diarrhoea containing blood, mucus, or neither; study done in Bangladesh; history or observation of bloody or mucoid stool used to indicate dysentery	412 (227)	298	55·1% (50·2–60·0)	85·0% (83·7–86·2)
Mo-Suwan et al (1979)^43^	1977	144 inpatients (age range not specified) with diarrhoea; study done in Thailand; observation of bloody stool by laboratory staff used to indicate dysentery	5 (2)	9	40·0% (5·3–85·3)	95·0% (89·9–98·0)

The appendix (p 3) summarises the associated QUADAS assessment. QUADAS=Quality Assessment of Diagnostic Accuracy Studies. WAZ=weight-for-age *Z* score.

* Dysentery of any cause.

† Severe malnutrition was defined using Gomez classification WAZ <60% of National Center for Health Statistics median.

From the systematic search of treatment, 882 titles and abstracts were reviewed, and 20 trials were included (figure 1). 17 (85%) were in children with dysentery, none were placebo-controlled, and ten (50%) evaluated antibiotics no longer recommended for acute infectious diarrhoea.^44^ Additionally, 14 (70%) were done in Asia (eight [40%] in Bangladesh), five (25%) in the Americas, and one (5%) was a multi-centre trial that included African and Asian sites (table 3). 12 (60%) trials were among children with dysentery and confirmed *Shigella* infection, six (30%) among children with dysentery (without *Shigella* confirmatory testing), and two (10%) among children with confirmed *Shigella* infection irrespective of dysentery status (but did not stratify by dysentery). All trials included a clinical outcome, such as clinical improvement or time to resolution of symptoms. 14 (70%) included a bacteriological outcome, such as bacteriological cure or time to negative stool culture. One (5%) study included mortality as an outcome. Of the 20 included trials, three (15%) compared different doses or durations of the same antibiotic, and 17 (85%) compared two different antibiotics, one (5%) of which also included a group treated with no antibiotic.^57^ One (5%) trial compared an antibiotic to supportive treatment, including the administration of an alternative antibiotic at the clinician's discretion.^60^ The quality of evidence for these included studies was very low (five [25%] of 20), low (eight [40%]), or moderate (seven [35%]; appendix p 4).

**Table 3:** Randomised controlled trials of antibiotic treatment for *Shigella* infections or dysentery, or both

	**Population and study characteristics**	**Intervention**	**Comparator**	**N**	**Outcomes of interest for systematic review**	**RR, HR, mean difference, or proportion of clinical cure (95% CI)**
Alam et al (1994)^45^	Inpatients aged 1–8 years with bloody diarrhoea lasting <72 h, >20 erythrocytes and pus cells per high power field, and culture-confirmed *Shigella* spp; study done in Bangladesh	Pivmecillinam 50 mg/kg per day for 5 days	Nalidixic acid 60 mg/kg per day for 5 days	71	Proportion with clinical improvement (≥1 formed stool without blood in the previous 24 h, with no fever [rectal temperature ≤37·8°C], and no abdominal pain or tenderness) on day 5; proportion with bacteriological cure on day 5	RR 1·42 (1·15–1·75); 1·25 (1·00–1·56)
Basualdo et al (2003)^46^	Inpatients aged 6 months to 5 years with dysenteric diarrhoea per physician's evaluation (≥2 bloody diarrhoeal stools in 24 h or the presence of >20 leucocytes per high power field on microscopy [or both], with fever, and abdominal pain or tenesmus [or both]) with culture-confirmed *Shigella* spp; study done in Paraguay	Azithromycin 12 mg/kg for 1 day, followed by 6 mg/kg per dose for 4 days	Cefixime 8 mg/kg per day for 5 days	75	Proportion with clinical cure (resolution or substantial improvement of signs and symptoms) at day 3; proportion with bacteriological cure at day 3	RR 1·19 (0·97–1·47); 0·72 (0·54–0·98)
Bhattacharya et al (1997)^47^	Inpatients aged 1–10 years with a history of acute bacillary dysentery (>3 stools in 24 h and passage of visible blood and mucus in stool for <3-day duration); study done in India	Norfloxacin 20 mg/kg per day in two divided doses for 5 days	Nalidixic acid 60 mg/kg in four divided doses for 5 days	22 had culture-confirmed *Shigella* spp	Mean duration of diarrhoea after therapy; mean duration of presence of blood in stool	2·7 days for norfloxacin group *vs* 3·7 days for nalidixic acid group (difference −1 day, −1·73 to −0·27); 1·4 days *vs* 2·4 days (−1, −1·58 to −0·42)
Dutta et al (1995)^48^	Inpatients aged <5 years diagnosed with dysentery (>3 loose stools per day, in which stool was intimately mixed with blood and mucus, and accompanied by symptoms: fever, abdominal pain, and tenesmus), of less than 3-day duration; patients who received treatments known to be effective against dysentery were excluded, as were children who had <10 bowel movements per day; study done in India	Furazolidone 7·5 mg/kg per day in four divided doses for 5 days	Nalidixic acid 55 mg/kg per day in four divided doses for 5 days	72	Clinical cure (no blood in stool, no fever, stool semi-solid with frequency <3 times for last 24 h or no stool for last 18 h) at day 5 of treatment	29 (85·3%) of 34 for furazolidone group *vs* 29 (100%) of 29 for nalidixic acid achieved clinical cure; p=0·039
Gilman et al (1980)^49^	Inpatient children with blood, pus cells, and mucus in stool, ≥4 stools per day, and culture-confirmed *Shigella* spp; study done in Bangladesh	Low dose ampicillin 50 mg/kg per day	Standard dose ampicillin 150 mg/kg per day	59 children	Mortality at day 21; proportion with microbiological failure on day 3	0 deaths occurred among 28 children in the low-dose group compared with 2 deaths among the 29 children in the high-dose group (risk difference −0·07, −0·02 to 0·02); 0 microbiological failures in either group on day 3
Gilman et al (1981)^50^	Inpatient adults and children aged 2–10 years passing blood and mucus in stools for <1 month, presence of faecal leucocytes, and culture-confirmed *Shigella* spp*;* study done in Bangladesh	Single-dose ampicillin 100 mg/kg	Multiple doses of ampicillin 100 mg/kg per day for 5 days	41	Proportion clinically failed (persistence of dysentery for 7 hospital days or its recurrence ≥7 days after initiation of therapy and a positive stool culture for *Shigella)* at day 21; proportion with bacteriological cure on day 21	RR undefined (risk difference 0·04 [95% CI −0·04 to 0·13]); RR 3·13 (0·38–25·6)
Helvaci et al (1998)^51^	Inpatients aged 1–13 years with acute bloody mucoid diarrhoea and culture-confirmed *Shigella* spp; study done in Bangladesh	Cefixime 8 mg/kg per day for 5 days	Ampicillin 100 mg/kg plus sulbactam 100 mg/kg three times a day for 5 days	65	Proportion with duration of fever between days 0 and 2; proportion with duration of diarrhoea between days 0 and 2; proportion with time to disappearance of blood in stool between days 0 and 2; mean duration spent in hospital	RR 1·46 (1·01–2·12); 3·56 (1·30–9·78); 2·80 (1·54–5·09); mean duration 3·4 days for the cefixime group *vs* 5·8 days for the ampicillin plus sulbactam group, difference −2·4 days (−3·20 to −1·60)
Islam et al (1994)^52^	Outpatients aged 1–8 years with bloody diarrhoea of <72 h duration and <20 pus cells per high power field via stool microscopy, and culture-confirmed *Shigella* spp; study done in Bangladesh	Gentamicin 30 mg/kg per day orally for 5 days	Nalidixic acid 60 mg/kg per day orally for 5 days	71	Proportion with clinical improvement (<6 stools without visible blood on day 5, with absence of fever [rectal temperature <37·8°C] and abdominal pain or tenderness) on day 5; proportion with bacteriological cure on day 5	RR 1·70 (0·85–3·39); 0·55 (0·34–0·87)
Moolasart et al (1999)^53^	Inpatients aged 6 months to 12 years with acute gastroenteritis (diarrhoea [≥3 loose stools or 1 bloody stool in a 24 h period] accompanied by fever, abdominal pain, or vomiting); study done in Thailand	Ceftibuten 9 mg/kg per day for 5 days	Norfloxacin 15 mg/kg per day for 5 days	8 had culture-confirmed *Shigella* infection	Time to clinical success (no definition given), in children infected with *Shigella*; proportion with microbiological cure at day 2, in those infected with *Shigella*	2·3 days for the ceftibuten group *vs* 2·0 days for the norfloxacin group, p>0·05 (NS); 100% for ceftibuten group *vs* 100% for norfloxacin group
Prado Camacho et al (1989)^54^	Outpatients aged 2–59 months with ≥3 watery stools during the preceding 24 h, lasting up to 5 days, and presence of polymorphonuclear leucocytes in the stool, in those who had received no treatments; study done in Mexico	Furazolidone 5 mg/kg per day in four divided doses for 5 days	Ampicillin 100 mg/kg per day in four divided doses for 5 days	78 (28 had culture-confirmed *Shigella* infection)	Proportion with treatment success at day 6 (absence of watery stools by day 5 plus a negative stool culture on day 6)	92·3% for the furazolidone group *vs* 51·3% for the ampicillin group, p=0·001
Prado et al (1992)^55^	Inpatient and outpatient children aged 6 months to 15 years presenting with bloody diarrhoea (grossly or by Haemoccult test) or diarrhoea with fever (≥38·5°C) and presence of faecal leucocytes in 1990 and in whom *Shigella* or enteroinvasive *Escherichia coli* was identified; study done in Guatemala and Argentina	Ceftibuten 4·5 mg/kg twice daily for 5 days	Co-trimoxazole (trimethoprim 5 mg/kg plus sulfamethoxazole 25 mg/kg) twice daily for 5 days	22	Mean duration of diarrhoea; mean duration of fever; microbiological cure 2 days after treatment	2·4 days in ceftibuten group *vs* 3·4 days in co-trimoxazole group (statistical significance not reported); 1·3 days *vs* 1·2 days (statistical significance not reported); 15·4% and 22·2% (statistical significance not reported); 2 patients in each group had *Shigella* isolated in stool after treatment
Prado et al (1993)^56^	Outpatient children aged 6 months to 13 years presenting with acute diarrhoea for <3 days, visible blood in stool, and presence of sheets of polymorphonuclear white cells on stool microscopic examination; or acute diarrhoea with presence of sheets of polymorphonuclear white cells on stool microscopic examination and a weight-for-height index >70% according to US National Center for Health Statistics standards; study done in Guatemala	Pivmecillinam 40 mg/kg per day in four divided doses for 5 days	Co-trimoxazole (5 mL twice per day in children <20 kg and 10 mL twice per day in children >20 kg) for 5 days	61 with culture-confirmed *Shigella*	Treatment failure (persistence of fever or visible blood in stool after 72 h of treatment); duration of isolation of *Shigella*, diarrhoea, fever, visible blood in stools, occult blood in stools, and pus cells in stools	5 (17%) of 29 in the pivmecillinam group had treatment failure *vs* 4 (13%) of 30 in the co-trimoxazole group (no statistical significance given); mean duration of diarrhoeal stools, faecal leucocytes, occult and visible blood, and isolation rates of *Shigella* were similar between treatment groups (statistical significance not reported)
Rodriguez et al (1989)^57^	Outpatients aged 2–59 months with passage of ≥3 watery stools in the last 24 h, history of diarrhoea up to 5 days before admission, and presence of polymorphonuclear leucocytes and blood in a stool sample; study done in Mexico	Co-trimoxazole (trimethoprim 8 mg/kg per day plus sulfamethoxazole 40 mg/kg per day) or furazolidone (7·5 mg/kg per day)	Supportive therapy: oral rehydration, antipyretics, or nutrition	125	Proportion with bacteriological cure (negative stool culture) on day 3; proportion with clinical cure (absence of diarrhoea and alleviation of all signs and symptoms) on day 3; proportion with treatment success (clinical cure on day 3 and bacteriological cure [if a pathogen was isolated] on day 6)	RR 1·14 (0·91–1·43) for co-trimoxazole group *vs* furazolidone group, 1·3 (0·9–1·87) for co-trimoxazole group *vs* control group, and 1·14 (0·78–1·68) for furazolidone group *vs* control group; RR 0·94 (0·80–1·11) for co-trimoxazole group *vs* furazolidone group, 1·82 (1·13–2·92) for co-trimoxazole group *vs* control group, and 1·93 (1·21–3·09) for furazolidone *vs* control group; RR 2·91 (1·33–6·39) for either antibiotic (co-trimoxazole or furazolidone group) *vs* control group
Salam et al (1988)^58^	Inpatients aged 6 months to 12 years with grossly visible bloody and mucoid diarrhoea assessed by stool specimen, lasting <72 h, and culture-confirmed *Shigella* spp; study done in Bangladesh	Nalidixic acid (55 mg/kg per day) for 5 days	Ampicillin (100 mg/kg per day) for 5 days	74	Proportion with clinical cure (no unformed stools and no fever [rectal temperature of ≥39°C]) on day 5; proportion with bacteriological cure on day 6	RR 1·05 (0·79–1·39); 1·00 (1·00–1·00)*
Salam et al (1998)^59^	Inpatients aged 2–15 years, with passage of grossly bloody mucoid stools for ≤72 h and culture-confirmed *Shigella* spp; study done in Bangladesh	Ciprofloxacin 10 mg/kg twice daily for 5 days	Pivmecillinam 15–20 mg/kg three times per day for 5 days	120	Proportion with clinical cure (absence of persistent dysentery by day 3 and ≤6 stools by day 6, with no bloody mucoid stools, ≤1 watery stool, and no fever [rectal temperature ≤37·8°C] on day 6; proportion with bacteriological cure on day 6; proportion with bloody mucoid stool >3 days in duration	RR 1·23 (0·98–1·54); 1·11 (1·02–1·20); 0·64 (0·30–1·37)
Taylor et al (1987)^60^	Community-based children between “a few months” and 5 years old with diarrhoea (≥3 loose stools with fever, vomiting, colic, or visibly bloody stool); study done in Thailand	Erythromycin 40 mg/kg per day in four divided doses for 5 days	Supportive treatment and co-trimoxazole (trimethoprim 8 mg/kg plus sulfamethoxazole 40 mg/kg) twice daily for 5 days if indicated by clinician	21 had *Shigella* infection	Proportion with diarrhoea at day 7, in children with *Shigella* spp initially isolated	38% for erythromycin group *vs* 14% for control group (statistical significance not reported)
Vinh et al (2000)^61^	Inpatients aged 3–14 years admitted with fever and bloody diarrhoea (>3 loose stools with obvious blood) between 1995 and 1999 with *Shigella* or enteroinvasive *Escherichia coli* identified in stool; study done in Vietnam	Daily nalidixic acid 55 mg/kg per day for 5 days	Ofloxacin 7·5 mg/kg twice daily for 1 day	66	Proportion with clinical cure (symptoms resolved and absence of new symptoms [relapse] within 5 days of treatment initiation; proportion with microbiological cure (absence of pathogen identified in stool sample from day 5)	75% for nalidixic acid group *vs* 90% for ofloxacin group (p=NS); 92% for nalidixic acid group *vs* 100% for ofloxacin group (p=NS)
Vinh et al (2011)^62^	Inpatients aged <15 years passing bloody or mucoid stools, with or without abdominal pain, tenesmus, or fever for <72 h before admission; study done in Vietnam	Gatifloxacin 10 mg/kg per day for 3 days	Ciprofloxacin 15 mg/kg twice daily for 3 days	494 (107 had *Shigella* infection)	Proportion with clinical failure (presence of fever [defined as ≥37·8°C], or persistence of vomiting, abdominal pain, or tenesmus with or without ≥3 loose stools with or without blood, mucus, or both) at day 5; proportion with bacteriological failure at day 3 or more; difference in time to diarrhoea clearance, measured in hours; difference in time to recovery from fever, measured in hours; difference in time to recovery from bloody diarrhoea, measured in hours	RR 1·35 (0·77–2·37); RR 0·66 (0·24–1·82); HR 0·98 (0·82–1·17); HR 1·00 (0·84–1·20); HR 1·11 (0·93–1·32)
Yunus et al (1982)^63^	Inpatient adults and children aged >3 months with symptoms of dysentery (blood in stool, abdominal pain, tenesmus, or fever), with culture-confirmed *Shigella* spp; study done in Bangladesh	Co-trimoxazole (trimethoprim plus sulfamethoxazole) 6 mg/kg per day every 12 h for 5 days	Ampicillin 50 mg/kg per day divided into doses every 6 h to patients >15 kg	118 (87 of whom were aged <15 years)	Time to negative culture; time to decline of fever; time to clearance of blood in stool; duration of persisting stool mucus; duration of abdominal pain	2·9 days for co-trimoxazole group *vs* 3·1 days for ampicillin group (p=NS); 1·3 days *vs* 1·5 days (p<0·01); 1·5 days *vs* 2·2 days (p<0·05); 3·9 days *vs* 4.9 days (p<0·01); 2·8 days *vs* 3·6 days (p<0·01)
Zimbabwe, Bangladesh, South Africa (Zimbasa) Dysentery study Group (2002)^64^	Inpatients aged 1–11 years passing stools with visible blood for ≤72 h; study done in Zimbabwe, Bangladesh, and South Africa	Short course ciprofloxacin 15 mg/kg every 12 h for 3 days; 2 days of placebo	Standard course ciprofloxacin 15 mg/kg every 12 h for 5 days	253	Proportion with treatment success (either resolution of illness [no bloody mucoid or watery stools and no more than a trace of blood in any stool, and ≤3 stools in the previous day] or marked improvement [no bloody mucoid stool and at most one watery stool and no more than a trace amount of blood]) at day 6; proportion with bacteriological cure at day 6	RR 0·94 (0·74–1·20); 1·00 (1·00–1·00)†

The appendix (p 4) summarises the associated GRADE quality assessment. RR=risk ratio. HR=hazard ratio. NS=non-significant. GRADE=Grading of Recommendations Assessment, Development and Evaluation.

* All patients in Salam et al (1988)^58^ were bacteriologically cured at day 6.

† All patients in the study by the Zimbabwe, Bangladesh, South Africa Dysentery study Group^64^ were bacteriologically cured at day 5.

**Figure 2:**
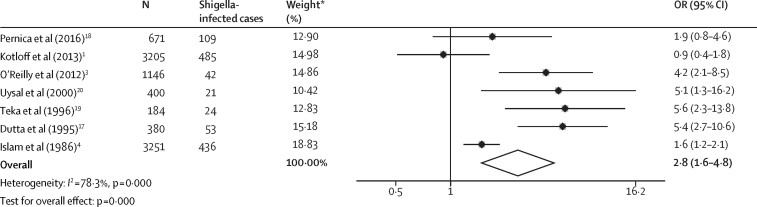
Individual and pooled effect estimate comparing the odds of death between children with and without laboratory-confirmed *Shigella* infection OR=odds ratio. *Weights are from random-effects analysis.

**Figure 3:**
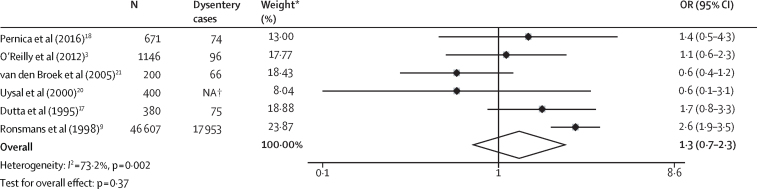
Individual and pooled effect estimates of studies comparing the odds of death in children with and without dysentery NA=not available. *Weights are from random-effects analysis. †Number of children with dysentery not reported.

Five (71%) of seven studies examining *Shigella* mortality relative to other causes of diarrhoea found the odds of death to be significantly higher in children with *Shigella* infection than in those without infection (figure 2, table 1). Substantial heterogeneity (*I*^2^=78·3%, p[*I*^2^]<0·001) was reported, with ORs ranging from 0·9 to 5·6. The random-effects pooled estimate suggested that *Shigella* infection was significantly associated with mortality (pooled OR 2·8, 95% CI 1·6–4·8; p=0·000). Six (46%) of 13 studies compared mortality in children with and without dysentery at diarrhoea presentation. Dysentery was defined as bloody stool (n=two), blood and mucus in stool (n=two), or no definition was described (n=two). Meta-analysis of included studies did not show an association between dysentery and mortality (pooled OR 1·3, 95% CI 0·7–2·3; p=0·37). Only one study reported a significant association (figure 3, table 1). There was substantial heterogeneity between dysentery mortality estimates (*I*^2^=73·2%, p[*I*^2^]=0·002). One study^21^ stratified *Shigella* mortality by the presence of dysentery but found no significant difference between inpatients with dysenteric *Shigella* and those with dysentery-negative *Shigella* in the association between *Shigella* infection and death. Four studies reported associations with death for *Shigella* and dysentery within the same population, and three of the four studies showed that *Shigella* infection had a significant association with death whereas dysentery had no association with death. A meta-analysis of these studies (appendix p 5) found *Shigella* infection to be significantly associated with mortality (OR 3·9, 95% CI 2·5–6·2, p=0·000; *I*^2^=18·3%, p(*I*^2^)=0·299) whereas dysentery was not (OR 1·3, 95% CI 0·9–2·0, p=0·20; *I*^2^=0%, p(*I*^2^)=0·636). The quality of evidence for the association between mortality and *Shigella* or dysentery was low to very low (appendix p 6).

Six studies reported inpatient case-fatality rates that were species specific, but none found *S dysenteriae* type 1 to be associated with a significantly higher inpatient case fatality than other species (table 4). No species-specific case-fatality rates for children who were not admitted to hospital were available. The quality of evidence for these rates was very low (appendix p 2) because of sparse and observational data. Too few mortality studies were identified to meaningfully test for publication bias.

**Table 4:** Studies of case-fatality rates associated with specific *Shigella* species

	**Enrolment dates**	**Population and study characteristics**	***Shigella dysenteriae* type 1**		**Other *S dysenteriae***		***Shigella flexneri***		***Shigella sonnei***		***Shigella boydii***	
			n/N	Case fatality (95% CI)	n/N	Case fatality (95% CI)	n/N	Case fatality (95% CI)	n/N	Case fatality (95% CI)	n/N	Case fatality (95% CI)
Bennish et al (1990)^10^	1974–88	Inpatients of all ages with diarrhoea and culture-confirmed *Shigella* spp; study done in Bangladesh; deaths ascertained during hospital admission for presenting diarrhoea	2221/9780	6·7% (5·3–7·8)	374/9780	8·2% (5·5–11·3)	6001/9780	10% (9·3–10·8)	445/9780	10·3% (7·7–13·5)	739/9780	8·4% (6·4–10·5)
Khan et al (2013)^6^	1987–89	Inpatients aged <15 years with diarrhoea and culture-confirmed *Shigella* spp; study done in Bangladesh; deaths ascertained during hospital admission for presenting diarrhoea	157/792	10·8% (6·4–16·8)	24/792	4·2% (0·1–21·1)	504/792	10·5% (8·0–13·5)	30/792	13·3% (3·8–30·7)	77/792	10·4% (0·1–19·0)
O'Reilly et al (2012)^3^	2005–07	Inpatients aged <5 years with watery, mucoid, or bloody diarrhoea; study done in Kenya; dysentery defined as visible bloody stool; *Shigella* spp detected by culture; deaths ascertained during hospital admission for presenting diarrhoea	..	..	4/42	50·0% (6·8–93·2)	30/42	23·3% (13·2–52·9)	6/42	16·7% (0·4–64·1)	2/42	100% (15·8–100)
van den Broek et al (2005)^21^	1993–99	Severely malnourished inpatients* aged <4 years with diarrhoea; study done in Bangladesh; unclear definition of dysentery; *Shigella* spp detected by culture; deaths ascertained during hospital admission for presenting diarrhoea	38/200	47·3% (31·0–64·2)	..	..	162/200	50·6% (42·6–58·6)	..	..	..	..
de Widerspach-Thor et al (2002)^65^	1996–97	All inpatients had culture-confirmed *Shigella* spp; study done in Djibouti; deaths ascertained during hospital admission for presenting diarrhoea	6/42†	16·7% (0·4–64·1)†	6/42†	16·7% (0·4–64·1)†	29/42	6·9% (0·8–22·8)	5/42	0% (0–52·2)	2/42	50·0% (12·6–98·7)
Zaman et al (1991)^66^	1978–87	All admissions had culture-confirmed *Shigella* spp; study done in Bangladesh; deaths ascertained during hospital admission for presenting diarrhoea	935/3440	0·9% (0·4–1·7)	..	..	1834/3440	1·1% (0·7–1·7)	..	..	..	..

Data are *Shigella* species (n)/total *Shigella* species (N). The appendix (p 2) summarises the associated GRADE quality assessment. 98 cases of other *S dysenteriae*, 194 *S sonnei*, and 379 *S boydii* are reported; however, no case-fatality rates are given for these serotypes in Zaman et al (1991).^66^ GRADE=Grading of Recommendations Assessment, Development and Evaluation. WAZ=weight-for-age *Z* score.

* Severe malnutrition was defined using Gomez classification WAZ <60% of National Center for Health Statistics median.

† All *S dysenteriae* cases combined.

**Figure 4:**
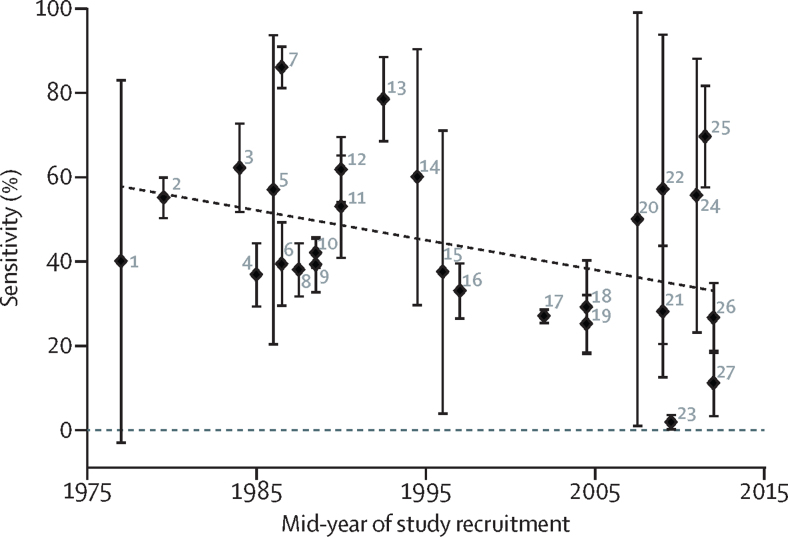
Sensitivity of dysentery for the detection of *Shigella* infection over time Error bars are 95% CI. Line of best fit is weighted to the inverse of the standard error for each estimate. Error bars are calculated by the Serrbar Stata package and therefore differ slightly to those displayed in table 2. 1=Mo-Suwan et al (1979).^43^ 2=Stoll et al (1982).^42^ 3=Ronsmans et al (1988).^9^ 4=Huskins et al (1994).^41^ 5=Moalla et al (1994).^40^ 6=Echeverria et al (1991).^39^ 7=Dutta et al (1992).^38^ 8=Kagalwalla et al (1992).^37^ 9=Ahmed et al (1997).^36^ 10=Khan et al (2013).^6^ 11=Sobel et al (2004).^35^ 12=Mathan et al (1991).^34^ 13=Nakano et al (1998).^33^ 14=Youssef et al (2000).^32^ 15=Suwatano et al (1997).^31^ 16=van den Broek et al (2005).^21^ 17=von Seidlein et al (2006).^13^ 18=Ozmert et al (2010).^30^ 19=Jafari et al (2008).^29^ 20=El-Shabrawi et al (2015).^28^ 21=Debas et al (2011).^27^ 22=Dooki et al (2014).^26^ 23=Hegde et al (2013).^25^ 24=Eseigbe et al (2013).^24^ 25=Aggarwal et al (2016).^23^ 26=Pernica et al (2016).^18^ 27=Pavlinac et al (2016).^22^

The sensitivity of dysentery for laboratory-confirmed *Shigella* infection ranged from 1·9% to 85·9% (table 2). Random-effects meta-regression showed that a significant amount of heterogeneity (p=0·04) was explained by a decreasing proportion of *Shigella* infections presenting with dysentery (sensitivity) over time (figure 4). Specificity had a narrower range of 64·4–100%, but there was no evidence of an association (p=0·60) between the absence of dysentery as a marker of *Shigella*'s absence (specificity) and time. 16 of the included studies were found to be of high quality (appendix p 3). 13 studies were downgraded for not offering clear definitions of dysentery. Four were downgraded because the indication for *Shigella* testing might have been influenced by the presence of dysentery. No evidence of publication bias was found in this analysis.

In the single trial that compared non-antibiotic supportive therapy with co-trimoxazole or furazolidone, antibiotic treatment had clinical and bacteriological benefit compared with no antibiotic treatment.^57^ In this study, the effect of antibiotics on clinical cure was strongest in children with *Shigella, Salmonella*, diarrhoeagenic *Escherichia coli*, or *Campylobacter* isolated at baseline (stratification by each bacteria was not reported). Three trials,^59^, ^62^, ^64^ specifically evaluated ciprofloxacin, the recommended treatment for dysentery by WHO. These trials reported equivalent clinical efficacy of ciprofloxacin compared with gatifloxacin^62^ or pivmecillinam,^59^ and a slightly higher bacteriological efficacy with ciprofloxacin than with pivmecillinam (table 2).^59^ One study^64^ found that treatment duration with ciprofloxacin (2-day short course *vs* 5-day long course) to have no effect on clinical or bacteriological efficacy. No differences were found for clinical and bacteriological outcomes in studies of co-trimoxazole versus pivmecillinam,^56^ co-trimoxazole versus ceftibuten (other than diarrhoea on day 4),^55^ ceftibuten versus norfloxacin,^53^ nalidixic acid versus ampicillin,^58^ nalidixic acid versus ofloxacin,^61^ and low-dose ampicillin versus standard-dose ampicillin.^49^ Additionally, no difference was seen between single-dose ampicillin and multiple doses of ampicillin.^50^ In one study,^46^ azithromycin was bacteriologically but not clinically superior to cefixime. Furazolidone was clinically superior to ampicillin^54^ in one study but inferior to nalidixic acid in another.^48^ Gentamicin was found to be bacteriologically but not clinically inferior to nalidixic acid;^52^ however, norfloxacin was clinically superior to nalidixic acid.^47^ One study showed that cefixime was clinically superior to ampicillin plus sulbactam,^51^ and another study showed that co-trimoxazole had better clinical, but not bacteriological, outcomes than ampicillin.^63^ Finally, one study^45^ reported that pivmecillinam was clinically and bacteriologically superior to nalidixic acid when cases with *Shigella* infections that are resistant to nalidixic acid were included.

## Discussion

Our systematic reviews found *Shigella* infection to be associated with mortality in children presenting with diarrhoea, and that dysentery did not adequately identify children with *Shigella* infections in many settings. Treatment strategies targeting dysentery-free *Shigella* infections might reduce diarrhoea-associated mortality. Because a range of antibiotics have shown efficacy in treating children with dysentery and *Shigella* infections, antibiotic treatment of high-risk groups of children without dysentery might be an effective addition to the current guidance.

The majority of *Shigella* mortality studies reported a significant association with death when compared with other causes of diarrhoea. The different populations, clinical management strategies, study designs, and enrolment periods resulted in marked heterogeneity in the magnitude of association across the studies. Most studies used standard culture to detect *Shigella* infection, with only one study using molecular methods,^18^ which can triple the detection rate by detecting lower-burden infections.^2^, ^67^ Length of follow-up and management practices also varied across studies with all but one study^1^ being limited to patients admitted to hospital without post-discharge follow-up. The prevalence of known risk factors for mortality (young age,^6^, ^10^ HIV infection,^68^ and severe acute malnutrition^6^, ^10^) varied across studies, and these subgroups of children might be at highest risk of *Shigella*-associated mortality. Despite these sources of heterogeneity, the pooled association of *Shigella* and mortality suggests that *Shigella* is an important risk factor for death in children with diarrhoea.

This meta-analysis found that *Shigella* infection had a stronger association with mortality than did dysentery. Only a single study, published in the 1980s, found a significant association between dysentery and mortality.^9^ However, children with *Shigella* infection and dysentery do have higher-burden infections.^2^ Also, *Shigella* dysentery, through its association with shiga-toxin production and very severe diarrhoea, is associated with severe complications such as haemolytic uraemic syndrome and severe hyponatraemia.^10^, ^69^ Given the established consequences of dysentery, the near absence of an association between dysentery and mortality in studies is likely to be the consequence of effective management strategies, including the administration of antibiotics.

Although *Shigella* infection was strongly associated with dysentery in all the included studies, dysentery was not a reliable tool for identifying *Shigella* infection. In other words, *Shigella* is common in children with dysentery, but most children infected with *Shigella* do not present with dysentery. The sensitivity of dysentery for identifying *Shigella* appears to have declined over time, although a subset of recent studies found dysentery to be fairly sensitive. Differences in the sensitivity of dysentery for *Shigella* infection across studies might be due to variability in *Shigella* species, such as the global decline in *S dysenteriae* type 1.^12^, ^13^ Declining prevalence of comorbidities such as malnutrition or measles could lead to populations being better equipped to mount an immune response to *Shigella* infection and, in turn, be less likely to develop dysentery after *Shigella* infection. Increased use of antibiotics might also play a role in declines of dysentery.

Most antibiotic trials for laboratory-confirmed *Shigella* infection were done among children with dysentery. Those that included children without dysentery did not report the treatment effect in non-dysenteric *Shigella* cases. Few differences were observed in clinical efficacy across a range of antibiotics tested, but these trials took place in different regions and across different eras, and some of the antibiotics evaluated might no longer be active against *Shigella* or are not readily available. The absence of placebo-controlled studies limits conclusions about the overall effectiveness of different antibiotics for the treatment of dysentery. However, given the accepted benefits of treating dysentery with antibiotics, placebo-controlled trials of dysenteric *Shigella* do not have equipoise. Because *Shigella* infection, irrespective of dysentery status, appears to be associated with death, antibiotics might play a role in the treatment of non-dysenteric *Shigella* infection. However, antibiotic resistance develops quickly in *Shigella* infections^70^ and will need to be weighed against increased antibiotic use. The development and use of a rapid diagnostic test for *Shigella* detection, and ideally a rapid diagnostic test for resistance to commonly used antibiotics, could be used to target treatment and minimise community-wide resistance.

This review had several limitations, most notably the heterogeneity in all analyses. This finding is unsurprising given the diverse populations, comorbidities, and *Shigella* species covered by this review. Many studies of dysentery epidemics were excluded because they presented only dysentery case fatality without a comparison population, prohibiting a calculation of an OR. These epidemic reports reinforce the importance of *S dysenteriae* type 1 to public health, documenting very high incidence and case-fatality rates of 1–11%.^71^ Data from South America and Africa were under-represented, which limits our findings' generalisability but highlights the need for further research in these regions. There was also substantial heterogeneity in the definition of dysentery. Most studies defined dysentery as bloody stool, but there was varied use of caregiver reports or provider observation for classification. Previous studies have shown that caregiver report of dysentery classifies up to five times more children as having dysentery than does laboratory-observed blood in stool.^22^ Mortality studies did not detail the causes of death, highlighting the need for highly characterised prospective cohorts to better understand mechanisms leading to death. Finally, included studies primarily used stool culture for *Shigella* identification, a less sensitive method than molecular methods.^2^ As a result, some children with *Shigella* infections could have been misclassified as not having *Shigella.* However, molecular techniques are unable to differentiate *Shigella* species and *Shigella*-like bacteria, such as enteroinvasive *E coli*, which complicates the attribution of diarrhoea and mortality to *Shigella*. These methods also do not have the ability to ascribe antimicrobial susceptibility patterns to individual pathogens, limiting the clinical use of molecular techniques.

In conclusion, *Shigella* infection is associated with an increased risk of mortality. Prevention of *Shigella* infections through vaccination or improvements in safe drinking water and sanitation will be the long-term solution to *Shigella*-associated mortality. In the meantime, effective *Shigella* identification and treatment strategies are needed. In most resource-limited settings, where bacterial culture is unavailable, reliance on dysentery for identifying children with *Shigella* might inadequately identify those at risk of death. Together, these findings suggest that clinicians should continue to aggressively manage dysentery, but should be aware that the absence of dysentery does not indicate a low risk of death and does not exclude *Shigella* as a cause of diarrhoea. It might be advisable to use pathogen-directed treatment when available, have a lower threshold for inpatient observation, or increase follow-up frequency in particularly vulnerable children with non-dysenteric diarrhoea, such as those younger than 2 years or those with malnutrition. There is an urgent need to reduce *Shigella*-associated morbidity and mortality, but the current evidence to support guideline development is inadequate and of low-to-moderate quality. Robust clinical trials to evaluate alternative interventional approaches to *Shigella* infection in children without dysentery are needed.

## References

[1] Kotloff KL, Nataro JP, Blackwelder WC (2013). Burden and aetiology of diarrhoeal disease in infants and young children in developing countries (the Global Enteric Multicenter Study, GEMS): a prospective, case-control study. Lancet.

[2] Liu J, Platts-Mills JA, Juma J (2016). Use of quantitative molecular diagnostic methods to identify causes of diarrhoea in children: a reanalysis of the GEMS case-control study. Lancet.

[3] O'Reilly CE, Jaron P, Ochieng B (2012). Risk factors for death among children less than 5 years old hospitalized with diarrhea in rural western Kenya, 2005–2007: a cohort study. PLoS Med.

[4] Islam SS, Shahid NS (1986). Morbidity and mortality in a diarrhoeal diseases hospital in Bangladesh. Trans R Soc Trop Med Hyg.

[5] Afroze F, Ahmed T, Sarmin M (2017). Risk factors and outcome of *Shigella* encephalopathy in Bangladeshi children. PLoS Negl Trop Dis.

[6] Khan WA, Griffiths JK, Bennish ML (2013). Gastrointestinal and extra-intestinal manifestations of childhood shigellosis in a region where all four species of *Shigella* are endemic. PLoS One.

[7] WHO (2005). The treatment of diarrhoea: a manual for physicians and other senior health workers.

[8] WHO (2005). Guidelines for the control of shigellosis, including epidemics due to *Shigella dysenteriae* type 1.

[9] Ronsmans C, Bennish ML, Wierzba T (1988). Diagnosis and management of dysentery by community health workers. Lancet.

[10] Bennish ML, Harris JR, Wojtyniak BJ, Struelens M (1990). Death in shigellosis: incidence and risk factors in hospitalized patients. J Infect Dis.

[11] Prince Christopher RH, David KV, John SM, Sankarapandian V (2010). Antibiotic therapy for *Shigella* dysentery. Cochrane Database Syst Rev.

[12] Livio S, Strockbine NA, Panchalingam S (2014). *Shigella* isolates from the global enteric multicenter study inform vaccine development. Clin Infect Dis.

[13] von Seidlein L, Kim DR, Ali M (2006). A multicentre study of *Shigella* diarrhoea in six Asian countries: disease burden, clinical manifestations, and microbiology. PLoS Med.

[14] Khatun F, Faruque AS, Koeck JL (2011). Changing species distribution and antimicrobial susceptibility pattern of *Shigella* over a 29-year period (1980–2008). Epidemiol Infect.

[15] Atkins D, Best D, Briss PA (2004). Grading quality of evidence and strength of recommendations. BMJ.

[16] Whiting P, Rutjes A, Johannes B, Bossuyt PM, Kleijnen J (2003). The development of QUADAS: a tool for the quality assessment of studies of diagnostic accuracy included in systematic reviews. BMC Med Res Methodol.

[17] Dutta P, Mitra U, Rasaily R (1995). Assessing the cause of in-patients pediatric diarrheal deaths: an analysis of hospital records. Indian Pediatr.

[18] Pernica JM, Steenhoff AP, Welch H (2016). Correlation of clinical outcomes with multiplex molecular testing of stool from children admitted to hospital with gastroenteritis in Botswana. J Pediatric Infect Dis Soc.

[19] Teka T, Faruque AS, Fuchs GJ (1996). Risk factors for deaths in under-age-five children attending a diarrhoea treatment centre. Acta Paediatr.

[20] Uysal G, Sokmen A, Vidinlisan S (2000). Clinical risk factors for fatal diarrhea in hospitalized children. Indian J Pediatr.

[21] van den Broek JM, Roy SK, Khan WA (2005). Risk factors for mortality due to shigellosis: a case-control study among severely-malnourished children in Bangladesh. J Health Popul Nutr.

[22] Pavlinac PB, Denno DM, John-Stewart GC (2016). Failure of Syndrome-based diarrhea management guidelines to detect *Shigella* infections in Kenyan children. J Pediatric Infect Dis Soc.

[23] Aggarwal P, Uppal B, Ghosh R, Krishna Prakash S, Chakravarti A, Rajeshwari K (2016). True prevalence of shigellosis in Indian children with acute gastroenteritis: have we been missing the diagnosis?. J Res Health Sci.

[24] Eseigbe EE, Iriah S, Ibok S (2013). Bacterial isolates from the stools of children aged less than 5 years with acute diarrhea in Kaduna, Northwestern Nigeria. Ann Trop Med Public Health.

[25] Hegde ST, Benoit S, Lopez B (2013). Burden of laboratory-confirmed shigellosis infections in guatemala 2007–2012: results from a population-based surveillance system. Am J Trop Med Hyg.

[26] Dooki MRE, Rajabnia R, Sawadkohi RB, Gatabi ZM, Poornasrollah M, Mirzapour M (2014). Bacterial entropathogens and antimicrobial susceptibility in children with acute diarrhea in Babol, Iran. Caspian J Intern Med.

[27] Debas G, Kibret M, Biadglegne F, Abera B (2011). Prevalence and antimicrobial susceptibility patterns of shigella species at Felege Hiwot Referral Hospital, Northwest Ethiopia. Ethiop Med J.

[28] El-Shabrawi M, Salem M, Abou-Zekri M (2015). The burden of different pathogens in acute diarrhoeal episodes among a cohort of Egyptian children less than five years old. Prz Gastroenterol.

[29] Jafari F, Shokrzadeh L, Hamidian M, Salmanzadeh-Ahrabi S, Zali MR (2008). Acute diarrhea due to enteropathogenic bacteria in patients at hospitals in Tehran. Jpn J Infect Dis.

[30] Ozmert EN, Orun E, Sengelen M, Yalcin SS, Yurdakok K, Gur D (2010). Sensitivity and specificity of bloody diarrhea in shigella gastroenteritis. Turk J Pediatr.

[31] Suwatano O (1997). Acute diarrhea in under five-year-old children admitted to King Mongkut Prachomklao Hospital, Phetchaburi province. J Med Assoc Thai.

[32] Youssef M, Shurman A, Bougnoux M, Rawashdeh M, Bretagne S, Strockbine N (2000). Bacterial, viral and parasitic enteric pathogens associated with acute diarrhea in hospitalized children from northern Jordan. FEMS Immunol Med Microbiol.

[33] Nakano T, Kamiya H, Matsubayashi N, Watanabe M, Sakurai M, Honda T (1998). Diagnosis of bacterial enteric infections in children in Zambia. Acta Paediatr Jpn.

[34] Mathan VI, Mathan MM (1991). Intestinal manifestations of invasive diarrheas and their diagnosis. Rev Infect Dis.

[35] Sobel J, Gomes TAT, Ramos RTS (2004). Pathogen-specific risk factors and protective factors for acute diarrheal illness in children aged 12–59 months in Sao Paulo, Brazil. Clin Infect Dis.

[36] Ahmed F, Clemens JD, Rao MR, Ansaruzzaman M, Haque E (1997). Epidemiology of shigellosis among children exposed to cases of *Shigella* dysentery: a multivariate assessment. Am J Trop Med Hyg.

[37] Kagalwalla AF, Khan SN, Kagalwalla YA, Alola S, Yaish H (1992). Childhood shigellosis in Saudi Arabia. Pediatr Infect Dis J.

[38] Dutta P, Bhattacharya SK, Sen D (1992). Shigellosis in children: a prospective hospital based study. Indian Pediatr.

[39] Echeverria P, Sethabutr O, Pitarangsi C (1991). Microbiology and diagnosis of infections with *Shigella* and enteroinvasive *Escherichia coli*. Rev Infect Dis.

[40] Moalla H, Fendri C (1994). Etiology of acute diarrhea in children. Tunis Med.

[41] Huskins WC, Griffiths JK, Faruque AS, Bennish ML (1994). Shigellosis in neonates and young infants. J Pediatr.

[42] Stoll BJ, Glass RI, Huq MI, Khan MU, Banu H, Holt J (1982). Epidemiologic and clinical features of patients infected with *Shigella* who attended a diarrheal disease hospital in Bangladesh. J Infect Dis.

[43] Mo-Suwan L, Varavithya W (1979). Clinical profile of diarrhoea at Ramathibodi Hospital during 1977. Southeast Asian J Trop Med Public Health.

[44] WHO (2017). WHO model lists of essential medicines. http://www.who.int/medicines/publications/essentialmedicines/en/.

[45] Alam AN, Islam MR, Hossain MS, Mahalanabis D, Hye HK (1994). Comparison of pivmecillinam and nalidixic acid in the treatment of acute shigellosis in children. Scand J Gastroenterol.

[46] Basualdo W, Arbo A (2003). Randomized comparison of azithromycin versus cefixime for treatment of shigellosis in children. Pediatr Infect Dis J.

[47] Bhattacharya K, Bhattacharya MK, Dutta D (1997). Double-blind, randomized clinical trial for safety and efficacy of norfloxacin for shigellosis in children. Acta Paediatr.

[48] Dutta P, Sett A, Sarkar A (1995). Comparative efficacy of furazolidone and nalidixic acid in the empirical treatment of acute invasive diarrhea: randomized clinical trial. Indian Pediatr.

[49] Gilman RH, Koster F, Islam S, McLaughlin J, Rahaman MM (1980). Randomized trial of high- and low-dose ampicillin therapy for treatment of severe dysentery due to *Shigella dysenteriae* type 1. Antimicrob Agents Chemother.

[50] Gilman RH, Spira W, Rabbani H, Ahmed W, Islam A, Rahaman MM (1981). Single-dose ampicillin therapy for severe shigellosis in Bangladesh. J Infect Dis.

[51] Helvaci M, Bektaslar D, Ozkaya B, Yaprak I, Umurtak B, Ertugrul A (1998). Comparative efficacy of cefixime and ampicillin–sulbactam in shigellosis in children. Acta Paediatr Jpn.

[52] Islam MR, Alam AN, Hossain MS, Mahalanabis D, Hye HK (1994). Double-blind comparison of oral gentamicin and nalidixic acid in the treatment of acute shigellosis in children. J Trop Pediatr.

[53] Moolasart P, Eampokalap B, Ratanasrithong M (1999). Comparison of the efficacy of ceftibuten and norfloxacin in the treatment of acute gastrointestinal infection in children. Southeast Asian J Trop Med Public Health.

[54] Prado Camacho JL (1989). A comparison of furazolidone and ampicillin in the treatment of invasive diarrhea. Scand J Gastroenterol Suppl.

[55] Prado D, López E, Liu H (1992). Ceftibuten and trimethoprim-sulfamethoxazole for treatment of *Shigella* and enteroinvasive *Escherichia coli* disease. Pediatr Infect Dis J.

[56] Prado D, Liu H, Velasquez T, Cleary TG (1993). Comparative efficacy of pivmecillinam and cotrimoxazole in acute shigellosis in children. Scand J Infect Dis.

[57] Rodriguez RS, Chavez AZ, Galindo E (1989). A randomized, controlled, single-blind study comparing furazolidone with trimethoprim–sulfamethoxazole in the empirical treatment of acute invasive diarrhea. Scand J Gastroenterol Suppl.

[58] Salam MA, Bennish ML (1988). Therapy for shigellosis. I. Randomized, double-blind trial of nalidixic acid in childhood shigellosis. J Pediatr.

[59] Salam MA, Dhar U, Khan WA, Bennish ML (1998). Randomised comparison of ciprofloxacin suspension and pivmecillinam for childhood shigellosis. Lancet.

[60] Taylor DN, Blaser MJ, Echeverria P, Pitarangsi C, Bodhidatta L, Wang WL (1987). Erythromycin-resistant *Campylobacter* infections in Thailand. Antimicrob Agents Chemother.

[61] Vinh H, Wain J, Chinh MT (2000). Treatment of bacillary dysentery in Vietnamese children: two doses of ofloxacin versus 5-days nalidixic acid. Trans R Soc Trop Med Hyg.

[62] Vinh H, Anh VTC, Anh ND (2011). A multi-center randomized trial to assess the efficacy of gatifloxacin versus ciprofloxacin for the treatment of shigellosis in Vietnamese children. PLoS Negl Trop Dis.

[63] Yunus M, Mizanur Rahman AS, Farooque AS, Glass RI (1982). Clinical trial of ampicillin v. trimethoprim-sulphamethoxazole in the treatment of *Shigella* dysentery. J Trop Med Hyg.

[64] Zimbabwe, Bangladesh, South Africa (Zimbasa) Dysentery Study Group (2002). Multicenter, randomized, double blind clinical trial of short course versus standard course oral ciprofloxacin for *Shigella dysenteriae* type 1 dysentery in children. Pediatr Infect Dis J.

[65] de Widerspach-Thor A, d'Alteroche L, Rault A, Thuan JF, Masseron T, Hovette P (2002). Predictive factors of mortality in severe shigellosis. Med Trop.

[66] Zaman K, Yunus M, Baqui AH, Hossain KM (1991). Surveillance of shigellosis in rural Bangladesh: a 10 years review. J Pak Med Assoc.

[67] Liu J, Kabir F, Manneh J (2014). Development and assessment of molecular diagnostic tests for 15 enteropathogens causing childhood diarrhoea: a multicentre study. Lancet Infect Dis.

[68] Keddy KH, Sooka A, Crowther-Gibson P (2012). Systemic shigellosis in South Africa. Clin Infect Dis.

[69] Bennish ML (1991). Potentially lethal complications of shigellosis. Rev Infect Dis.

[70] Gu B, Zhou M, Ke X (2015). Comparison of resistance to third-generation cephalosporins in *Shigella* between Europe–America and Asia–Africa from 1998 to 2012. Epidemiol Infect.

[71] Bennish ML, Wojtyniak BJ (1991). Mortality due to shigellosis: community and hospital data. Rev Infect Dis.

