# Supplementary material for: Identification and management of Shigella infection in children with diarrhoea: a systematic review and meta-analysis
Source: Lancet Glob Health. 2017 Nov 10;5(12):e1235–48. doi: 10.1016/S2214-109X(17)30392-3 (PMC5695759; doi:10.1016/S2214-109X(17)30392-3)

THE LANCET
Global Health

Supplementary appendix

This appendix formed part of the original submission and has been peer reviewed. We post it as supplied by the authors.

Supplement to: Tickell KD, Brander RL, Atlas HE, Pernica JM, Walson JL, Pavlinac PB. Identification and management of Shigella infection in children with diarrhoea: a systematic review and meta-analysis. *Lancet Glob Health* 2017; **5:** e1235–48.

Appendix I: Search Criteria and variables collected.

Search: Shigella/dysentery-associated mortality

Terms: titles and abstracts containing the terms dysentery, bacillary dysentery, shigellosis, or *Shigella* and mortality, death, or fatality were considered for full text review.

Variable collected: publication date, dates of enrollment, country, study population, study design, total number of subjects included, number of patients with dysentery, number with laboratory-confirmed *Shigella*, number of dysentery deaths, number of *Shigella* deaths, effect estimates (odds ratios [OR], relative risks [RR], or hazard ratios [HR] and 95% confidence intervals [CI]) describing risk of death associated with dysentery or *Shigella*, and species-specific effect estimates when available.

Search: Predictive value of dysentery for identifying Shigella

Terms: Titles and abstracts containing the terms dysentery, bacillary dysentery, shigellosis, or *Shigella* and identification, diagnosis or sensitivity/specificity were considered for possible inclusion.

Variables: publication date, dates of enrollment, country, study population, study design, total number of subjects included, number of subjects with dysentery, number with laboratory-confirmed *Shigella*, sensitivity of dysentery for detecting laboratory-confirmed *Shigella*, and the specificity of dysentery absence for identifying children without laboratory-confirmed *Shigella*.

Search: Treatment of Shigella/dysentery

Terms: Titles and abstracts containing the terms antibiotic, antiinfective, anti-infective, antimicrobial, antiparasitic, anti-parasitic, antiprotozoa*, anti-protozoa*, ciprofloxacin, erythromycin, or metronidazole, and "bloody stool", diarrh*, dysentery, *Shigella*, or gastroenterit*, and clinical trial, placebo-controlled trial randomized controlled trial, but not cancer or antibiotic associated diarrhea were evaluated.

Filters: Clinical Trial; Humans; Child: birth-18 years

Variables: publication date, dates of enrollment, country, study population, study design, total number of subjects included, intervention treatment, comparisons treatment, outcome(s), and estimate of benefit.

Appendix II: Study Quality Assessments

Shigella/dysentery-associated mortality

Methods**:** Evidence was assessed using the Grading of Recommendations Assessment, Development and Evaluation (GRADE) approach. In summary, all studies were awarded 2 points, instead of 4 points, due to being observational rather than randomized studies. Single points were deduced for sparse data (< 200 participants), lack of description of *Shigella* detection or dysentery determination methods, having a more than 5% loss to follow-up, or using an indirect population ("direct" defined as those only including children presenting to health services with diarrhea). Quality criteria applicable only to randomized trials were not applied (e.g. blinding, allocation concealment). Studies were then categorized as high quality (4 points), moderate (3 points), low (2 points), or very low (1 point) based on their final score.

Appendix II Table 1. Summary of GRADE quality assessment of studies evaluating the odds of death associated with culture confirmed *Shigella* spp. or dysentery at diarrhea presentation as compared to children without *Shigella* infection or dysentery

Full Citation	Shigella Mortality Assessment		Dysentery Mortality Assessment		Directness		Final score	
	Sparse data (<200)	Describes method of *Shigella* diagnosis	Loss to follow >5%	Sparse data (<200)	Describes dysentery definition	Loss to follow >5%		
**Bennish 1990**	0	0	0	--	--	--	Indirect: Includes adults	Very low
**De Widerspach-Thor 2002**	-1	0	0	--	--	--	Indirect: Includes adults	Very low
**Dutta 1995**	-1	0	0	-1	0	0	Direct: Admitted children	Very low
**Islam 1986**	0	0	0	--	--	--	Indirect: Includes adults	Very low
**Khan 2013**	0	0	0	--	--	--	Direct: Admitted children	Low
**Kotloff 2013**	0	0	0	--	--	--	Direct: Recruited at presentatio n	Low
**O'Reilly 2012**	-1	0	0	-1	0	0	Direct: Admitted children	Very low
**Pernica 2015**.	-1	0	0	-1	0	0	Direct: Admitted children	Very low
**Ronsmans 1988**	--	--	--	0	0	0	Indirect: Community identification	Very low
**Teka 1996**	-1	0	0	--	--	--	Direct: Admitted children	Very low
**Uysal 2000**	-1	-1	0	-1	-1	0	Direct: Admitted children	Very low
**Van den Broek 2005**	--	--	--	-1	-1	0	Indirect: Severely malnourish ed children	Very low
**Zaman 1991**	0	0	0	--	--	--	Indirect: Includes adults	Very low

Predictive value of dysentery for identifying Shigella

Methods**:** Quality was assessed using the Quality Assessment of Diagnostic Accuracy Studies (QUADAS) criteria assuming dysentery was the diagnostic test being evaluated in included studies. Specific operationalized definitions of QUADAS quality assessment indicators are included as footnotes. No score was determined as was recommended in the QUADAS methodology.

Appendix II Table 2. Summary of QUADAS quality assessment of studies evaluating the sensitivity and specificity of dysentery at diarrhea presentation for the identification of *Shigella*-infected children

**Full Citation**	**QUADAS Assessment**													
	Representative patients[1]	Clear selection criteria[2]	Accurate reference standard[3]	Appropriate time-period[4]	Universal application of reference test[5]	Received same reference test despite index test[6]	Index not part of reference[7]	Index test defined[8]	Reference test defined[9]	Index interpret without reference[10]	Reference interpret without index[11]	Clinical data available[12]	Uninterpretable results reported[13]	Withdrawal explained[14]
**Aggarwal 2016**	N	N	Y	Y	Y	Y	Y	Y	Y	Y	Un	Y	Y	Y
**Ahmed 1997**	Y	Y	Y	Y	Y	Y	Y	Y	Y	Y	Un	Y	Y	Y
**Debas 2011**	N	Y	Y	Y	Y	Y	Y	Y	Y	Y	Un	Y	Y	Y
**Dooki 2014**	N	Y	Y	Y	Y	Y	Y	N	Y	Y	Un	Y	Y	Y
**Dutta 1992**	Y	Y	Y	Y	Y	Y	Y	Y	Y	Y	Un	Y	Y	Y
**Echeverria 1991**	Un	N	Un	Un	Un	Un	Un	N	N	N	Un	Y	Y	Y
**El-Shabrawi 2015**	Y	Y	Y	Y	Y	Y	Y	Y	Y	Y	Un	Y	Y	Y
**Eseigbe 2013**	Y	Y	Y	Y	Y	Y	Y	N	Y	Y	Un	Y	Y	Y
**Hegde 2013**	N	Y	Y	Y	N	N	Y	N	N	Y	Un	Y	Y	Y
**Huskins 1994**	Y	Y	Y	Y	Y	Y	Y	N	N		Un	Y	Y	Y
**Jafari 2008**	Y	Y	Y	Y	Y	Y	Y	Y	Y	Y	Un	Y	Y	Y
**Kagalwalla 1992**	Y	Y	Y	Y	Y	Y	Y	N	Y	Y	Un	Y	Y	Y
**Khan 2013**	Y	Y	Y	Y	Y	Y	Y	N	N	Y	Un	Y	Y	Y
**Mathan 1991**	N	Y	Y	Y	Y	N	Y	N	N	Un	Un	Y	Y	Y
**Moalla 1994**	Y	Y	Y	Y	Y	Y	Y	N	Y	Y	Un	Y	Y	Y
**Mo-Suwan 1979**	Y	Y	Y	Y	Y	Y	Y	Y	N	N	Un	Y	Y	Y
**Nakano 1998**	Y	Y	Y	Y	Y	Y	Y	N	Y	Y	Un	Y	Y	Y
**Ozmert 2010**	Un	Y	Y	Y	Y	Un	Un	N	N	Un	Un	Y	Y	Y
**Pavlinac 2015**	Y	Y	Y	Y	Y	Y	Y	Y	Y	Y	Un	Y	Y	Y
**Pernica 2015.**	Y	Y	Y	Y	Y	Y	Y	Y	Y	Y	Un	Y	Y	Y
**Ronsmans 1988**	N	Y	Y	Y	Y	Y	Y	Y	Y	Y	Un	Y	Y	Y
**Sobel 2004**	Y	Y	Y	Y	Y	Y	Y	N	Y	Y	Un	Y	Y	Y
**Stoll 1982**	Y	Y	Y	Y	Y	Y	Y	Y	Y	Y	Un	Y	Y	Y
**Suwatano 1997**	Y	Y	Y	Y	Y	Y	Y	N	Y	Y	Un	Y	Y	Y
**Van den Broek 2005**	N	Y	Y	Y	Y	Y	Y	Y	Y	Un	Un	Y	Y	Y
**Von Seidlein 2006**	Y	Y	Y	Y	Y	Y	Y	Y	Y	Y	Un	Y	Y	Y
**Youssef M, 2000**	Y	Y	Y	Y	Y	Y	Y	Y	Y	Y	Un	Y	Y	Y

[1]Representative patients: Children under 18 years old presenting to health services. Active case finding in the community was not considered representative. [2] Clear selection Criteria: Selection criteria given in methods. [3]Accurate reference standard: Both culture and molecular methods were considered accurate. [4]Appropriate time-frame: Samples and observations made during the same diarrheal illness. [5]Universal application of reference test: everyone included got reference test. [6]Received same reference test despite index test: Reference not conditional on result of index test. [7]Index not part of reference: Index not used as part of the reference test. [8]Index test defined: Dysentery defined, including the use of blood or blood & mucus in stool, or whether caregiver report, provider observed or laboratory confirmed blood in stool was used. [5] Index interpret without reference: Dysentery result obtained without knowledge of the culture/molecular results. [9]Reference test defined: Culture or molecular techniques described in reasonable detail. [11] Reference interpret without index: Culture or molecular results obtained without dysentery status being known. [12] Clinical data available: Clinical

data was comparable to normal practices during interpretation (i.e. no extra diagnostics tests performed). [13] Uninterpretable results reported: No uninterpretable tests possible. 14Withdrawal explained: Were any participants who were consented not included in the analysis.

Treatment of Shigella/dysentery

Methods. Evidence was assessed using a modified Grading of Recommendations Assessment, Development and Evaluation (GRADE) approach. In summary, all studies were awarded 4 points as all were randomized control trials. One point was deducted for each of the following elements: <200 participants included in the trial, >5% loss to follow up or withdrawals, or lack of double-blinding. No additional points were awarded or deducted for consistency or effect size because of heterogeneity in interventions tested and outcomes assessed. We did not include the GRADE components of directness because only trials from children with dysentery or *Shigella* were included. Studies were then categorized as high quality (4 points), moderate (3 points), low (2 points), or very low (1 point) based on their final score.

Appendix II Table 3. Summary of GRADE quality assessment of randomized controlled trials of antibiotic treatment for *Shigella* infections and/or dysentery antibiotics. A bibliography of included papers, and associated GRADE quality assessments, are included in this paper's supplementary materials.

**Table 4 Citations**	**Modified GRADE Assessment**			
**Full Citation**	**Sparse data (<200)**	**Loss to follow or withdrawals >5%**	**Not double-blind**	**Final modified score**
**Alam 1994**	-1	0	0	Moderate
**Basualdo 2003**	-1	-1	-1	Very low
**Bhattacharya 1997**	-1	0	0	Moderate
**Dutta 1995**	-1	-1	-1	Very low
**Gilman 1980**	-1	0	-1	Low
**Gilman 1981**	-1	0	-1	Low
**Helvaci 1998**	-1	0	0	Moderate
**Islam 1994**	-1	0	0	Moderate
**Moolasart 1999**	-1	-1	-1	Very low
**Prado Camacho 1989**	-1	-1	-1	Very low
**Prado 1993**.	-1	0	0	Moderate
**Prado 1992**	-1	0	-1	Low
**Rodriguez 1989**	-1	0	-1	Low
**Salam 1988**	-1	0	0	Moderate
**Salam 1998**	-1	-1	0	Low
**Taylor 1987**	-1	0	-1	Low
**Vinh 2011**	0	-1	-1	Low
**Vinh 2000**	-1	-1	-1	Very low
**Yunus 1982**	-1	0	-1	Low
**Zimbabwe, Bangladesh, South Africa (Zimbasa) Dysentery Study Group 2002**	0	-1	0	Moderate

Appendix III: Supplementary Figures

Appendix III Figure 1. The individual and pooled odds ratios of studies which compared both the odds of death between children with and without laboratory -confirmed *Shigella* infection (A) and the odds of death between children with and without dysentery (B).

A: Shigella estimates

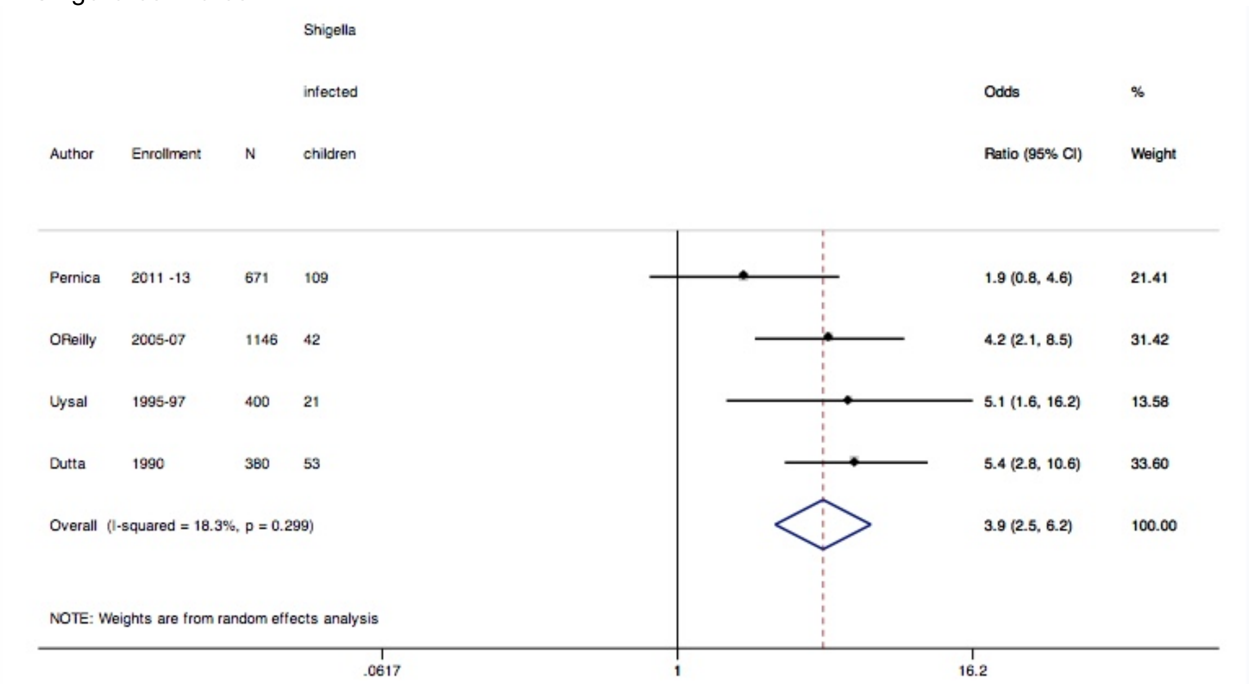

B: Dysentery estimates

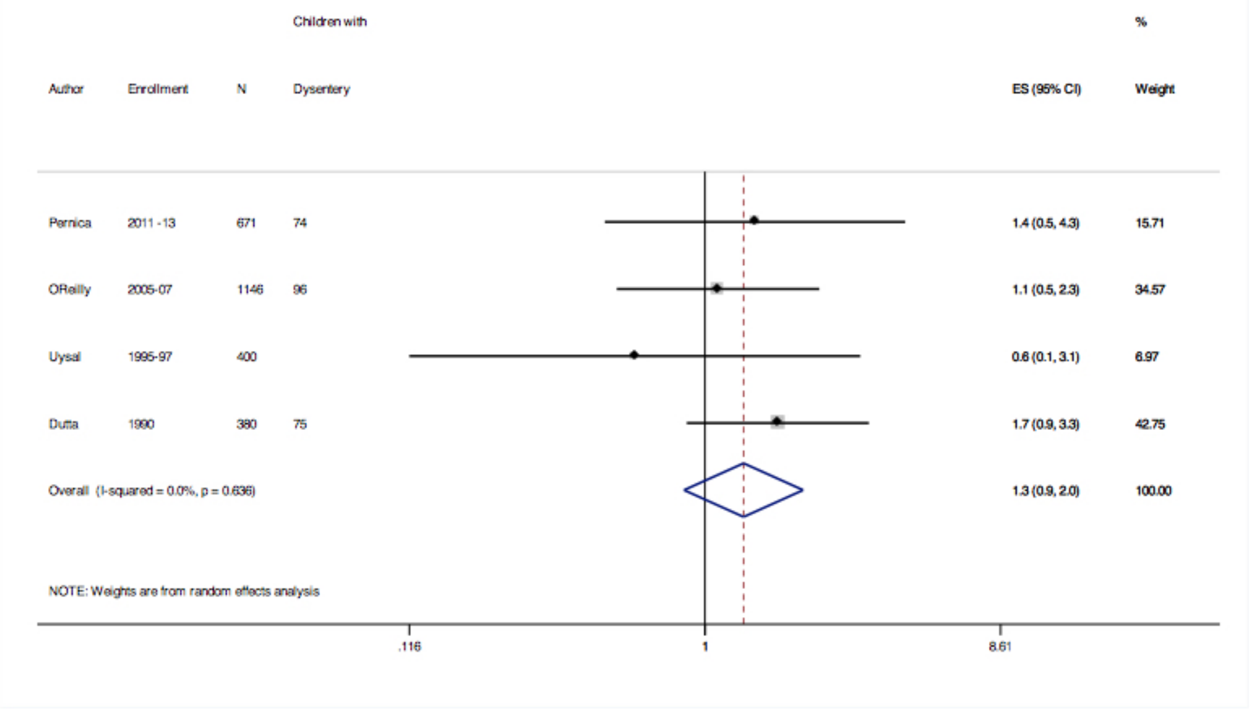

Appendix III Figure 2. Funnel plots for assessment of potential publication bias for studies evaluating the association between *Shigella* and mortality (A), the association between dysentery and mortality (B), the sensitivity of dysentery for identifying confirmed *Shigella* (C), and the specificity of dysentery- the absence of dysentery indicating the absence of *Shigella* (D)

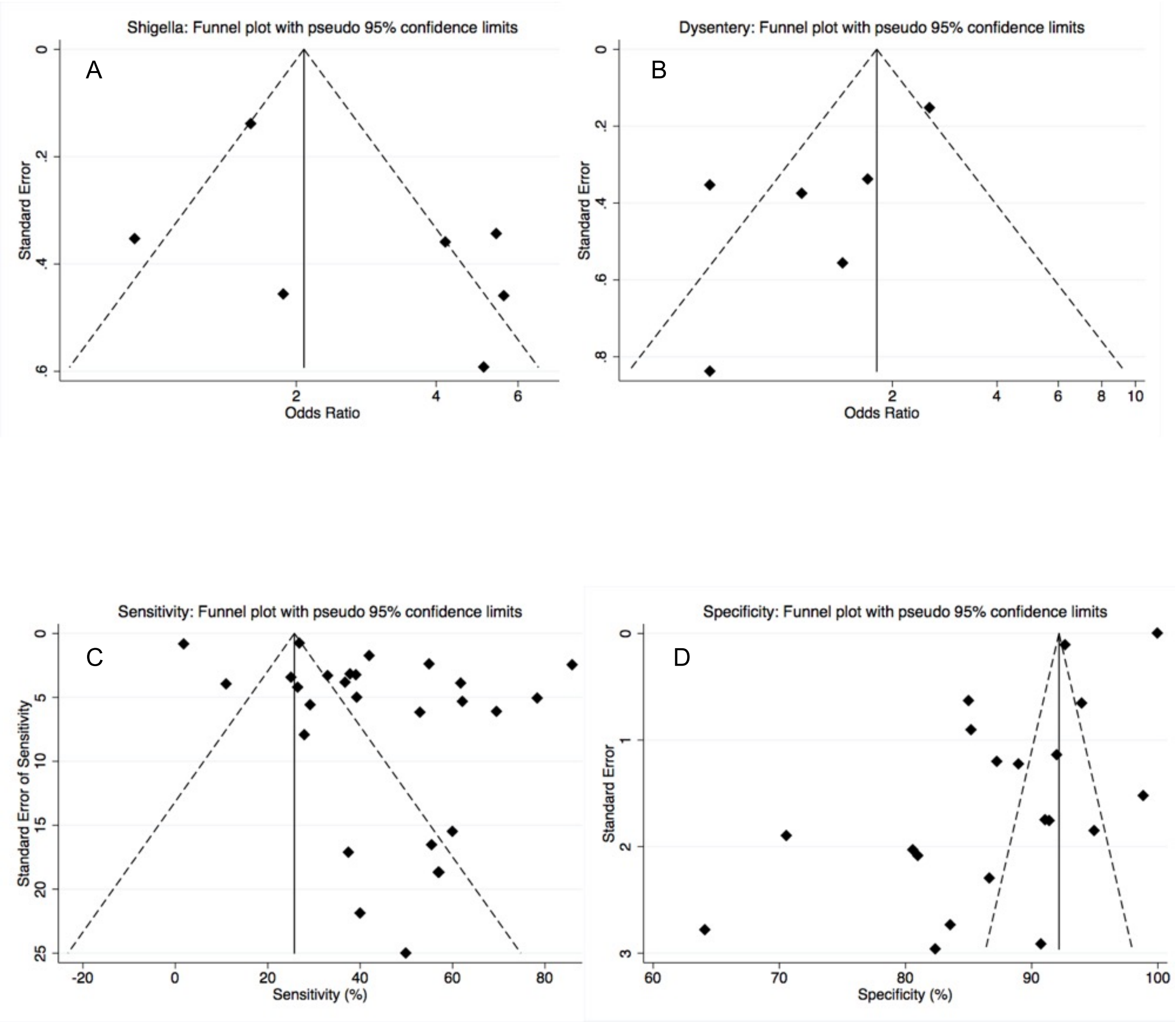

Supplement: Supplementary appendix [file mmc1.pdf]
